# Diagnostic Challenges and Outcome of Classical Phenylketonuria in a Resource-Constrained Middle Eastern Country

**DOI:** 10.1155/ijpe/5246296

**Published:** 2025-08-29

**Authors:** Nadine Yazbeck, Rudy N. Zalzal, Fadi El Oueichek, Jinane Samaha, Abir Barhoumi, Pascale E. Karam

**Affiliations:** ^1^Division of Gastroenterology and Nutrition, Department of Pediatrics and Adolescent Medicine, American University of Beirut Medical Center, Beirut, Lebanon; ^2^Faculty of Medicine, American University of Beirut, Beirut, Lebanon; ^3^Department of Nutrition, American University of Beirut Medical Center, Beirut, Lebanon; ^4^Inherited Metabolic Diseases Program, Department of Pediatrics and Adolescent Medicine, American University of Beirut Medical Center, Beirut, Lebanon

## Abstract

**Background:** Scarce data on classical phenylketonuria diagnosis and outcome in low-income Middle Eastern countries is available. The effect of phenylketonuria diet on growth parameters is still controversial. This 15-year retrospective study is aimed at examining the diagnosis, outcome, and growth of classical phenylketonuria patients following a phenylalanine-restricted Mediterranean diet in Lebanon.

**Methods:** A retrospective review of the charts of patients diagnosed and followed between 2008 and 2023 at the American University of Beirut Medical Center, Lebanon, was conducted. Age at diagnosis, molecular profile, neurological status, anthropometric measurements, diet, and metabolic control were analyzed.

**Results:** Out of 82 patients, 35 met the inclusion criteria. The majority were late-diagnosed (average age: 4 years) with poor neurological outcome. The Mediterranean variant IVS10-11G>A in a homozygous state was identified in 63%. There was no statistically significant difference between body mass index or height for age *z*-scores at first and last encounter for all patients on phenylalanine-restricted Mediterranean diet.

**Conclusions:** Phenylalanine-restricted Mediterranean diet seems to preserve growth parameters in classical phenylketonuria patients. However, achieving a neurotypical outcome remains challenging in the absence of systematic newborn screening in Lebanon, a resource-constrained country with high rates of consanguinity.

## 1. Introduction

Classical phenylketonuria (PKU) (OMIM 261600) is an autosomal recessive metabolic disorder due to phenylalanine (Phe) hydroxylase deficiency resulting in hyperphenylalaninemia. It is the most common inborn error of metabolism with variable prevalence depending on geographic regions and ethnicities, occurring mostly in populations with high consanguinity rates. PKU has an estimated prevalence of 1 in 23,930 live births worldwide [1]. The highest prevalence was reported in the Middle East, mainly in Turkey (38.13/100,000) [2]. In Lebanon, consanguinity reaches 26% [3] with a PKU prevalence estimated at 14.29/100,000 neonates [4].

PKU may be classified into three types, based on the plasma Phe level at diagnosis: classical PKU defined as Phe levels > 1200 *μ*mol/L, whereas mild and benign PKU are considered with Phe levels of 600–1200 *μ*mol/L and 120–600 *μ*mol/L, respectively [5]. Diagnosis is confirmed by molecular identification of disease-causing variants in the PAH gene [6]. Classical PKU is currently defined by an initial pretreatment blood Phe level above 1200 *μ*mol/L, a low dietary Phe tolerance (less than 500 mg/day) [7], and a corresponding genotypic profile [6].

In high-income countries, the early detection of PKU is based on neonatal screening; however, in resource-constrained countries, most patients are still diagnosed when symptomatic at variable ages [8]. Early diagnosis and treatment aiming to lower plasma Phe levels are crucial [9]. Patients diagnosed by newborn screening and treated before 10 days of life are expected to achieve good neurological and intellectual outcomes [5]. Untreated PKU may lead to neurological complications including intellectual disability, autism, psychomotor delay, and seizures of variable severity [10]. Treatment relies mainly on a hypercaloric diet with Phe restriction and an obligatory supplementation of essential amino acids mixture without the offending amino acid. This dietary management aims to maintain plasma Phe levels below a “nontoxic” range, variably defined across metabolic disease centers worldwide [11]. For children under 12 years of age, the recommended target blood Phe is 120–360 *μ*mol/L, while acceptable levels after 12 years of age can go up to 600 *μ*mol/L [12]. Additional therapies may also be used, mainly the synthetic form of Phe hydroxylase cofactor, sapropterin hydrochloride, in responsive patients [9]. Such a diet can affect growth patterns in classical PKU patients. Sena et al. [13] reported overweight and obesity in treated children and adolescents with PKU, whereas Robertson et al. [14] found that growth outcomes were similar to those of the general population. The debate remains ongoing, with controversial reports in the literature. Furthermore, there is limited data on the diagnosis and outcome of classical PKU in resource-constrained Middle Eastern countries [8, 15].

In this 15-year retrospective study, we report the diagnostic challenges and outcome of classical PKU patients diagnosed and followed at the Inherited Metabolic Diseases Program at the American University of Beirut Medical Center, the referral center for inborn errors of metabolism in Lebanon [4].

## 2. Material and Methods

A retrospective review of the charts of all sequential patients diagnosed with classical PKU and followed at the reference center for Inherited Metabolic Diseases of the American University of Beirut Medical Center, Beirut, Lebanon, between 2008 and 2023 was conducted. This retrospective study was approved by the Institutional Review Board at the American University of Beirut, Lebanon, Protocol Number: BIO-2018-0381.

Clinical presentation, age at diagnosis and genetic profile, anthropometric measurements, and neurological outcome were studied. Metabolic control was evaluated by calculating the average blood Phe concentration for each patient throughout the follow-up duration period.

The analyzed sample included all patients with classical PKU diagnosed and followed during the study period. Classical PKU was defined by the biochemical, phenotypic, and genotypic triad: initial plasma Phe level above 1200 *μ*mol/L, dietary Phe tolerance less than 500 mg/day, and a corresponding genotypic profile. Patients born prematurely, and/or suffering from chronic diseases, and/or treated with sapropterin hydrochloride or any other adjuvant medication were excluded. Patients with incomplete charts and/or lost to follow-up within 6 months after diagnosis were also excluded.

Demographic data including sex at birth and consanguinity were noted. The studied variables were age at diagnosis and diagnostic modality (detection by newborn screening or upon clinical manifestations), as well as genetic diagnosis (zygosity and identified variant in the Phe hydroxylase gene) for each patient. In addition, the neurological status, anthropometric measurements, and metabolic control at diagnosis before dietary therapy initiation and at last assessment were also analyzed. All studied patients were diagnosed and followed by the same experienced specialized metabolic and nutrition team.

### 2.1. Neurological Status

The neurological presentation and outcome were analyzed. Neurological assessment was performed by a multidisciplinary team including two experienced pediatricians (a metabolic diseases specialist and a pediatric gastroenterologist), a pediatric neurologist, and a child psychiatrist, ensuring comprehensive assessment of neurological, cognitive, and behavioral functions. Neurological evaluation comprised clinical examination, electroencephalography, brain magnetic resonance imaging, and administration of age-appropriate Wechsler intelligence scales as well as adaptive behavior assessment system when needed. The presence of motor delay and/or seizures at diagnosis and at last assessment was also recorded. Intellectual disability was categorized as mild, moderate, or severe, based on the intellectual and adaptive functioning.

### 2.2. Anthropometric Measurements

Anthropometric measurements were studied at diagnosis and at last encounter. For children under 2 years of age, weight was measured on a regularly calibrated infant scale (with a maximum of 20 kg) without clothes on except for a clean diaper. Length was measured in the supine position using a rigid pediatric height rod with a measuring range of 0–100 cm and an accuracy of 0.1 cm. Children above 2 years of age were weighed on a regularly calibrated scale barefoot. Height was measured in the standing position using a stadiometer with a measuring range of 85–200 cm and an accuracy of 0.1 cm. A trained pediatric nurse carried out all the measurements. The body mass index (BMI) value was the product of dividing the weight and squared height. The BMI *z*-score was calculated with Anthro (Version 3.2.2.1, Geneva, Switzerland) for patients from 0 to 5 years old and AnthroPlus software (Version 1.0.4., Geneva, Switzerland) for individuals from 5 to 19 years old. Based on BMI *z*-score, patients were classified as underweight for a value less than −1, normal weight between −1 and 1, overweight more than 1, and obese more than 2. Length/height for age *z*-score between −2 and +2 was considered normal, whereas stunting was defined by a value less than 2 [16].

### 2.3. Metabolic Control

Treatment for all classical PKU patients was exclusively based on dietary intervention with a hypercaloric, Phe-restricted diet and Phe-free essential amino acid mixture supplementation, along with vitamins and minerals supplementation. Caloric needs were met by adding a carbohydrate- and fat-rich medical product. Mean Phe tolerance, based on serial dietary recalls by the same dietitian, and the average plasma Phe levels throughout the follow-up period were calculated for each patient. For patients below 1 year of age, Phe levels were checked on filter paper weekly and monthly by plasma amino acids chromatography. For older patients, plasma amino acids were performed every 3 months and blood Phe level on filter paper monthly.

### 2.4. Statistical Analysis

The statistical analyses were performed with SPSS 28.0 (SPSS Inc., Chicago, IL). BMI and height *z*-scores were presented as means. Paired sample *t*-tests were used to assess changes in the mean within each of the two groups. The reported *p* values are two-sided.

The effect of a Phe-restricted diet on height and BMI was evaluated using McNemar's test for paired categorical data to assess changes in growth *z*-scores between age at diagnosis and age at last follow-up. Height and BMI *z*-scores were dichotomized into normal and abnormal categories based on WHO criteria. The reported *p* values are two-sided. The level of significance was set at *p* < 0.05.

## 3. Results

A total of 82 patients diagnosed with classical PKU were identified during the study period; however, 47 patients were excluded due to incomplete charts, and/or loss to follow-up within 6 months, and/or prematurity and/or treatment with adjuvant medications and/or chronic diseases. Thirty-five sequential patients from 35 families diagnosed with classical PKU were included in this study: 19 females (54%) and 16 males (46%). Most patients (80%) were offspring of consanguineous parents.

### 3.1. Age at Diagnosis and Diagnostic Modality

The majority (57%) were diagnosed beyond the neonatal period between 2 months and 13 years of age (average 4 years and 3 months) upon clinical manifestations (Table 1). Less than half of the patients (43%) were identified by newborn screening during the neonatal period (Table 2). Follow-up duration varied between 6 months and 14 years and 7 months.

### 3.2. Genetic Diagnosis

All patients carried homozygous pathogenic variants in the Phe hydroxylase gene. The “Mediterranean variant” NM_000277.3(PAH): c.1066-11G>A (IVS10-11G>A) was the most common allele identified in 63% of the patients, followed by c.473G>A (p.Arg158Gln) in 14%.

### 3.3. Neurologic Status

The majority of the “late-diagnosed” patients (75%) presented with intellectual disability, while 45% had motor delay and 25% suffered from seizures (Table 1). All patients detected by newborn screening were asymptomatic at diagnosis (Table 2). Age at last follow-up varied between 6 months and 19 years (average: 7 years and 2 months). Almost all late-diagnosed patients (90%) suffered from variable degrees of intellectual disability (Table 1). Seizures persisted in affected patients, while motor delay was reversible in two patients (M11 and M12) diagnosed before 1 year of age. Patients diagnosed by neonatal screening had normal neurological outcomes and displayed neurotypical behavior, except for a 9-year-old patient who had learning difficulties (Table 2).

### 3.4. Anthropometric Measurements

Anthropometric indices including weight, height, and *z*-scores for BMI and height for age were analyzed for all patients at diagnosis and at last visit. There was no statistically significant difference between the BMI *z*-scores or height for age *z*-scores at initial encounter compared to last visit (Table 3). At last assessment, 31.4% of all PKU patients were overweight and 25.7% obese, irrespective of their age at diagnosis.

**Figure 1:**
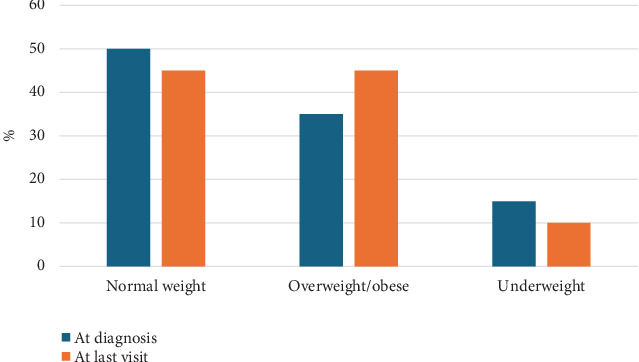
Body mass index *z*-score of “late-diagnosed” classical phenylketonuria patients at diagnosis and at last visit. Normal weight: between −1 and 1, overweight: more than 1, obese: more than 2, underweight less than −1.

In the late-diagnosed group, half of the patients had initially normal BMI *z*-score, 35% were overweight/obese, and 15% were underweight; at last follow-up, the majority remained in the same category (Figure 1). Moreover, they had a normal height *z*-score at initial and last encounter. McNemar's test did not demonstrate a statistically significant change in the proportion of patients classified as having high BMI over time (*χ*^2^ = 0.25, *p* = 0.62). An exact binomial test yielded consistent results (two-tailed *p* = 0.625). These findings indicate no significant overall shift in weight status following PKU dietary intervention. Similarly, there was no statistically significant change in the prevalence of short stature (height *z*‐score <–2) from age at diagnosis to last follow-up, indicating no measurable effect of PKU diet on height status over time (McNemar's test, *χ*^2^(1) = 0.5, *p* = 0.48).

**Figure 2:**
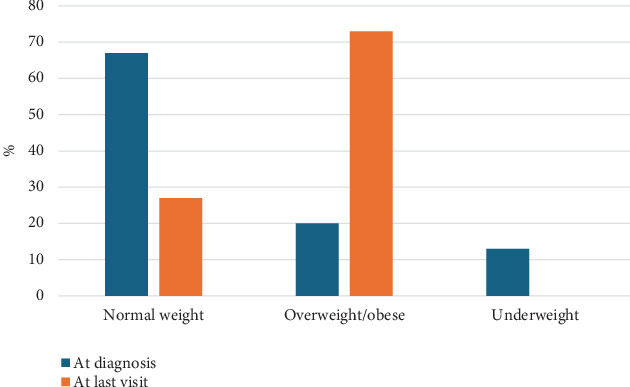
Body mass index *z*-score of classical phenylketonuria patients detected by newborn screening at diagnosis and at last visit. Normal weight: between −1 and 1, overweight: more than 1, obese: more than 2, underweight less than −1.

Most patients (73%) detected by newborn screening became overweight/obese at last follow-up (Figure 2). Length for age *z*-score was normal at diagnosis in 60%, whereas at last follow-up, all patients reached a normal length/height for age *z*-score. McNemar's test confirmed a statistically significant increase in the proportion of children with high BMI over time (*χ*^2^ = 5.14, *p* = 0.023). An exact binomial test confirmed this finding (two-tailed *p* = 0.016). These results indicate a significant upward shift in BMI classification over the follow-up period. In addition, there was a statistically significant improvement in height status among patients from diagnosis to last follow-up, with a reduction in short stature prevalence over time (*χ*^2^(1) = 4.17, *p* = 0.041).

Adolescent patients, aged 10–19 years as defined by WHO, were analyzed as a distinct subgroup, as this population is known to exhibit higher rates of dietary nonadherence and an elevated risk of overweight and obesity [17, 18]. There was no significant change in height (*χ*^2^ = 1.0, *p* = 0.317) nor in BMI *z*-scores (*χ*^2^ = 0.2, *p* = 0.654) over time (Table 3).

Another subgroup including patients who were nonadherent to the diet was analyzed. There was no statistically significant change in overweight status (BMI *z*‐score > +1; *p* = 0.25) or short stature status (height *z*‐score <–2; *p* = 1.00) between diagnosis and final follow-up (Table 4).

### 3.5. Metabolic Control

All 35 patients followed the Mediterranean, Phe-restricted diet; their mean Phe tolerance below 12 years of age was 340 and 462 mg/day afterwards. The average plasma Phe pretreatment level was 1525 *μ*mol/L for late-diagnosed patients and 432 *μ*mol/L during the follow-up period, whereas in the group detected by newborn screening, it was 1289 *μ*mol/L at diagnosis and 331 *μ*mol/L during the follow-up period (Table 2).

## 4. Discussion

The outcome of classical PKU patients has drastically improved with the implementation of newborn screening for early detection and therapy initiation [5]. In resource-constrained countries with high consanguinity rates and high prevalence of severe classical PKU, the diagnosis, treatment, and outcomes of this autosomal recessive disorder remain challenging. In this study, a total of 35 patients from 35 families were studied. Most patients were diagnosed upon neurological manifestations, while only 43% were screened and identified during the neonatal period. Although newborn screening for PKU was made available in Lebanon almost three decades ago, first by Guthrie test, then by expanded tandem mass spectrometry since 2007 [4, 19], the lack of a national policy for neonatal screening impeded its implementation across the country. Even when babies are screened and detected at birth, access to proper treatment is hindered by the scarcity of inherited metabolic disease physicians and specialized dieticians in Lebanon, as well as the urban location of the referral center, increasing the expenses incurred by patients living in remote areas.

The genotypic profile of the studied Lebanese patients with classical PKU revealed a predominance of the Mediterranean variant, NM_000277.3(PAH): c.1066-11G>A, IVS10-11G>A in 63% of the patients. This intronic variant was reported in 5.3% worldwide [6]; however, in Mediterranean countries, it was the most common biallelic genotype reported, reaching a frequency of 18.4% in Iran, followed by Turkey (13.9%) and Israel (9.4%) [1]. Interestingly, all studied Lebanese patients carried homozygous biallelic variants, reflecting the high rates of consanguinity in the population. The severity of the neurological sequelae reflected the genotype–phenotype correlation observed in PKU [6].

Untreated patients (diagnosed after 7 years of age) or those late-diagnosed (between 3 months and 7 years) may suffer from variable motor or cognitive delays [20]. More than half of the patients in this study were late-diagnosed after 3 months of age, with 30% after 7 years of age, which likely contributed to the poor neurological outcome observed in variable degrees among these patients. On the other hand, patients diagnosed and treated in the neonatal period displayed a neurotypical outcome so far, similarly to other reports [2].

Studies of the impact of the Mediterranean diet on anthropometric measurements and growth are scarce [21]. To date, the evidence of the impact of PKU on growth is still inconsistent. In a systematic review and meta-analysis, Ilgaz et al. [22] reported that children with PKU were significantly shorter and had lower weight for age than reference populations during the first 4 years of life. The impaired linear growth was still present until the end of adolescence. In a cross-sectional study conducted in Taiwan, Weng et al. [23] did not find any significant difference in height, weight, and BMI between PKU patients and healthy controls. On the other hand, Ahmadzadeh et al. report a high rate (44%) of overweight and obesity among Iranian PKU patients [21].

In Lebanon, no recent national data is available on overweight and obesity status for children less than 19 years. Few reports suggest overweight and obesity percentages of 33% and 13%, respectively [16, 24]. In the early-diagnosed classical PKU patients, higher weight gain *z*-scores observed may be explained by the compliance to the high-caloric fat-rich dietary intake in addition to the medical hypercaloric formula. However, the anthropometrics *z*-score data between first and last encounters did not differ significantly for all studied classical PKU patients irrespective of the age at diagnosis and diet initiation. The Lebanese patient population consumes by default the Mediterranean diet, which is plant-based, abundant in fruits, vegetables, and cereals, among others, relying on olive oil as the primary source of fat. It is also characterized by low to moderate dairy intake and limited amounts of meat and eggs. Therefore, adjusting the dietary prescription to accommodate PKU guidelines/restrictions does not bring a major change in our population dietary habits. The inclusion of a higher amount of the oil (reaching naturally 40% of total energy component) and being generous with fruits/vegetables, low by default in Phe, may have preserved the limited amount of natural protein to be exclusively spared for tissue build-up and repair, hence leading to unchanged growth patterns in classical PKU patients.

This study is limited by its retrospective nature. The results may not represent all the classical PKU patients in Lebanon; other unscreened patients may not have been referred to our center in view of their nonspecific neurological presentation and lack of specialized metabolic centers in remote areas of the country.

## 5. Conclusions

Scarce studies on the diagnosis and outcome of classical PKU in Middle Eastern resource-constrained countries are available. This is the first study from Lebanon reporting the diagnostic challenges, neurological outcome, and the dietary restriction effect on growth parameters in classical PKU patients. This study highlights the need for national policies for systematic newborn screening in resource-constrained countries with a high prevalence of PKU. The Phe-restricted Mediterranean diet, naturally low in protein and rich in healthy fats, seems to preserve the growth parameters in classical PKU patients.

## Figures and Tables

**Table 1:** Diagnosis and outcome of “late-diagnosed” classical phenylketonuria patients in the referral tertiary care center in Lebanon.

**Patient**	**Initial plasma Phe (*μ*mol/L)**	**Average plasma Phe ** ^**a**^ ** (*μ*mol/L)**	**At diagnosis**						**At last assessment**						**Genetic diagnosis**
			**Age**	**Anthropometrics**		**Neurological presentation**			**Age**	**Anthropometrics**		**Neurological outcome**			**PAH gene variant (homozygous)**
				**Height ** **z** ** -score**	**BMI ** **z** ** -score**	**Intellectual disability**	**Motor delay**	**Sz**		**Height ** **z** ** -score**	**BMI ** **z** ** -score**	**Intellectual disability**	**Motor delay**	**Sz**	
M1	1797	622	10 years	−2.26	−0.12	Severe	+	+	19 years	−2.78	1.69	Severe	+	+	c.842C>T
M2	1400	539	10.5 years	0.53	3.04	Mild	−	−	15.5 years	−0.02	1.57	Mild	−	−	c.1066-11G>A
M3	1980	386	4 years	0.71	1.7	Mild	−	−	14 years	−0.16	2.71	Mild	−	−	c.1066-11G>A
M4	1167	486	5 years	0.07	0.6	Severe	−	+	17 years	0.24	−2.01	Severe	−	+	c.473G>A
M5	1477	544	4.5 years	−2	1.75	Moderate	−	−	12 years	−2.10	0.71	Moderate	−	−	c.1066-11G>A
M6	1009	325	6 months	−0.53	−0.27	Mild	−	−	3.5 years	−0.72	−4.10	Mild	−	−	c.1066-11G>A
M7	1100	508	16 months	−1.83	0.15	Mild	−	−	10 years	0.68	1.98	Mild	−	−	c.143T>C
M8	1715	758	13 years	−0.17	−1.37	Moderate	−	−	19 years	−0.48	−0.11	Moderate	−	−	c.1066-11G>A
M9	1161	414	3.3 years	−1.37	4.02	Moderate	−	−	18 years	−2.43	1.05	Moderate	−	−	c.165del
M10	1579	554	3 years	−0.21	0.99	Moderate	−	−	11 years	−0.16	2.08	Moderate	−	−	c.473G>A
M11	1107	299	9 months	−2.62	0.17	−	+	−	6.8 years	−2.18	−0.88	Mild	−	−	c.1066-11G>A
M12	1924	354	4 months	−0.19	2.01	−	+	−	9 years	0.16	2	Mild	−	−	c.1066-11G>A
M13	3318	407	9 years	0.4	−0.09	Severe	+	+	10 years	−0.11	−0.5	Severe	+	+	c.1066-11G>A
M14	1582	315	4 months	−4.28	−3.17	−	+	−	10 months	−3.65	0.53	−	+	−	c.1066-11G>A
M15	1088	278	9 years	0.57	0.33	Severe	+	+	10 years	−0.11	−0.17	Severe	+	+	c.1222C>T
M16	1322	347	4 months	−2.49	−1.78	−	+	+	8 months	−2.1	−0.91	−	+	+	c.1066-11G>A
M17	1730	623	10 years	0.97	2.44	Severe	+	−	12 years	0.55	2.6	Severe	+	−	c.1066-11G>A
M18	1130	281	4 months	−1.47	−0.77	−	−	−	3.8 years	−1.50	−0.14	Mild	−	−	c.1066-11G>A
M19	1322	288	18 months	1.13	2.16	Mild	−	−	2 years	−0.48	2.22	Mild	−	−	c.1066-11G>A
M20	1594	328	23 months	−1.98	0.17	Severe	+	−	3.4 years	−1.34	0.30	Severe	+	−	c.1066-11G>A

Abbreviations: m, months; Sz, seizures; y, years.

^a^During the follow-up period.

**Table 2:** Diagnosis and outcome of classical phenylketonuria patients detected by neonatal screening in the referral tertiary care center in Lebanon.

**Patient**	**Initial plasma Phe**	**Mean plasma Phe**	**At diagnosis**		**At last assessment**			**Genetic diagnosis**
			**Height ** **z** ** -score**	**BMI ** **z** ** -score**	**Age**	**Height ** **z** ** -score**	**BMI ** **z** ** -score**	**PAH gene variant (homozygous)**
M21	1701	348	−0.27	0.87	4 years	0.23	2.68	c.1222C>T
M22	1078	284	0.26	0.46	7 years	0.86	0.19	c.1222C>T
M23	1305	416	2.79	2.16	12 years	0.83	2.77	c.473G>A
M24	1579	359	−4.64	−0.76	5 years	−0.87	3.46	c.473G>A
M25	1076	428	−0.32	−1.02	3 years	0.33	2.54	c.165del
M26	1204	331	−1.51	0.46	2 years	0.19	0.14	c.1066-11G>A
M27	1466	296	0.17	−0.03	2 years	1.87	1.85	c.1066-11G>A
M28	1211	357	−2.88	−0.16	4 years	−1.73	1.5	c.1066-11G>A
M29	1029	286	−2.12	−0.49	6 months	−0.33	1.9	c.1066-11G>A
M30	1702	319	−0.34	1.57	6 months	−0.45	1.09	c.1066-11G>A
M31	1320	356	0.17	−0.3	7 months	0.36	0.01	c.1066-11G>A
M32	1148	252	1.21	−0.14	1 year	−0.58	−1	c.1066-11G>A
M33	1217	238	−2.37	0.03	16 months	0.14	1.35	c.842C>T
M34	1004	357	−2.22	1.02	9 years and 2 months⁣^∗^	−0.47	4.24	c.473G>A
M35	1300	288	−2.42	−3.38	2 years	−1.91	1.09	c.1066-11G>A

Abbreviations: m, months; y, years.

^a^Learning difficulties.

**Table 3:** Anthropometric parameters of adolescent patients at diagnosis and last follow-up. Age 1: age at diagnosis; Age 2: age at last follow-up.

	**Age 1**	**Height ** **z** ** -score**	**BMI ** **z** ** -score**	**Age 2**	**Height ** **z** ** -score**	**BMI ** **z** ** -score**
M1	10 years	−2.26	−0.12	19 years	−2.78	1.69
M2	10.5 years	0.53	3.04	15.5 years	−0.02	1.57
M3	4 years	0.71	1.7	14 years	−0.16	2.71
M4	5 years	0.07	0.6	17 years	0.24	−2.01
M5	4.5 years	−2	1.75	12 years	−2.10	0.71
M8	13 years	−0.17	−1.37	19 years	−0.48	−0.11
M9	3.3 years	−1.37	4.02	18 years	−2.43	1.05
M10	3 years	−0.21	0.99	11 years	−0.16	2.08
M17	10 years	0.97	2.44	12 years	0.55	2.6
M23	1 month	2.79	2.16	12 years	0.83	2.77

**Table 4:** Anthropometric parameters of noncompliant patients at diagnosis and last follow-up. Age 1: age at diagnosis; Age 2: age at last follow-up.

**Patient**	**Age 1**	**Height ** **z** ** -score**	**BMI ** **z** ** -score**	**Age 2**	**Height ** **z** ** -score**	**BMI ** **z** ** -score**
M1	10 years	−2.26	−0.12	19 years	−2.78	1.69
M2	10.5 years	0.53	3.04	15.5 years	−0.02	1.57
M4	5 years	0.07	0.6	17 years	0.24	−2.01
M5	4.5 years	−2	1.75	12 years	−2.10	0.71
M7	16 months	−1.83	0.15	10 years	0.68	1.98
M8	13 years	−0.17	−1.37	19 years	−0.48	−0.11
M10	3 years	−0.21	0.99	11 years	−0.16	2.08
M17	10 years	0.97	2.44	12 years	0.55	2.6

## Data Availability

The data that support the findings of this study are available from the corresponding author upon reasonable request.
